# 3D Printing for Soft Tissue Regeneration and Applications in Medicine

**DOI:** 10.3390/biomedicines9040336

**Published:** 2021-03-26

**Authors:** Sven Pantermehl, Steffen Emmert, Aenne Foth, Niels Grabow, Said Alkildani, Rainer Bader, Mike Barbeck, Ole Jung

**Affiliations:** 1 Clinic and Policlinic for Dermatology and Venereology, University Medical Center Rostock, 18057 Rostock, Germany; sven.pantermehl@med.uni-rostock.de (S.P.); steffen.emmert@med.uni-rostock.de (S.E.); aenne.foth@med.uni-rostock.de (A.F.); ole.jung@med.uni-rostock.de (O.J.); 2 Institute for Biomedical Engineering, University Medical Center Rostock, 18119 Rostock, Germany; niels.grabow@uni-rostock.de; 3 BerlinAnalytix GmbH, 12109 Berlin, Germany; saidkildani@berlinanalytix.com; 4 Clinic and Policlinic for Orthopedics, University Medical Center Rostock, 18057 Rostock, Germany; rainer.bader@med.uni-rostock.de; 5 Department of Ceramic Materials, Chair of Advanced Ceramic Materials, Institute for Materials Science and Technologies, Technical University Berlin, 10623 Berlin, Germany

**Keywords:** 3D bioprinting, additive manufacturing, tissue engineering, regenerative medicine, soft tissue

## Abstract

The use of additive manufacturing (AM) technologies is a relatively young research area in modern medicine. This technology offers a fast and effective way of producing implants, tissues, or entire organs individually adapted to the needs of a patient. Today, a large number of different 3D printing technologies with individual application areas are available. This review is intended to provide a general overview of these various printing technologies and their function for medical use. For this purpose, the design and functionality of the different applications are presented and their individual strengths and weaknesses are explained. Where possible, previous studies using the respective technologies in the field of tissue engineering are briefly summarized.

## 1. Introduction

Since the first applications were developed in the 1980s, additive manufacturing (AM) has rapidly found its way into a wide variety of production processes in regenerative medicine. An essential component of AM technologies is 3D-printing. The first such technology was stereolithography (SLA), which was followed by the rapid development of various 3D printing applications, each with its own strengths, weaknesses, and areas of application. Nowadays, these technologies are mainly used in the manufacturing industry, which also includes the production of medical applications such as implants, orthotics, or anatomical models. A great advantage of 3D printing is the relatively quick and cost-effective production of materials for a wide range of possible usage.

The 3D printing technologies available today already offer the possibility of creating precise and individual constructs, tools, or models in a comparatively short time. In the health sector in particular, however, this technology may only be just beginning. With the prospect of producing entire tissues or organs using 3D printing in the future, all large parts of medicine could be entirely revolutionized.

A very important use of these technologies in modern medicine is characterized by the term “tissue engineering” or “bioprinting”. In this area, the disciplines of medicine, biology, mechanical engineering, materials science, and stem cell research overlap with the aim of using the benefits of 3D printing technology for medicine, and ultimately, the health of the individual. The production of artificial tissues from human cells could make it possible to improve the research on a wide range of diseases as well as possible therapeutic options, entirely without the need for additional animal testing, and thus with results that reflect the immediate reactions in human tissue.

Another exceptionally important application is the point of organ donation. Eurotransplant statistics from 2019 showed that the 13,985 organs transplanted in that year were nevertheless matched by a waiting list of over 7000 in need of organs [1]. In addition, there are possible rejection complications of transplanted organs and the disadvantages of lifelong immunosuppression of the transplanted persons. The possibility of simply “printing” the required organs directly from stem cells taken from the patient could possibly solve both problems at once.

The following review provides a basic overview of available 3D printing applications, their functionality as well as their strengths and weaknesses. The reader should be given an understandable and clear introduction to the various 3D printing technologies and their possible future impacts in the medical field. Therefore, not only were the applications suitable for use in bioprinting, in its actual definition, considered, but all 3D printing technologies were analyzed for their potential suitability for soft tissue regeneration.

For this purpose, the different literature research databases such as PubMed, Scopus, or ScienceDirect were searched and, where possible, exemplary studies using the respective 3D printing technology, especially in the field of soft tissue printing, were added to the explanations of the individual techniques.

The aim of this review was to provide newcomers to the field of additive manufacturing with an introduction to the subject and at the same time, offer more experienced researchers a basis for their own new ideas or approaches to conduct their own studies.

## 2. Working Stages of 3D Printing

**Figure 1:**
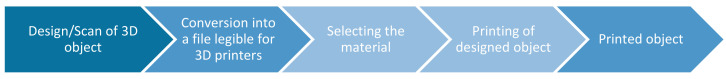
Working stages in 3D printing.

The basic principle of 3D printing is almost the same for all applications (Figure 1) and ultimately only differs in the technical design. The following four steps are part of the basic workflow of all applications:

1. The first step involves designing or scanning the object to be printed. The necessary scan is usually done via Computed Tomography (CT) or Magnetic Resonance Imaging (MRI). Alternatively, the required objects can also be created completely digitally using computer aided design (CAD) software. This allows each building, or, in the case of bioprinting, each tissue to be printed according to the required mechanical properties and structure, or to the patient’s requirements and thus optimally adapted to the individual pre-existing anatomical structures [2].
2. The resulting 3D model is now digitally converted into several horizontal 2D layers. Each individual layer contains the necessary data that the printer will later need for creating the desired object layer by layer [3,4]. How this merging is done in detail differs for each printer application.
3. The next step is to select the right material for printing, depending on the properties of the required building. It should be noted that different materials not only have different effects on the object in question depending on their composition, viscosity or mechanical strength, but can often only be printed by certain printing applications [5].
4. The last step includes the actual printing process. Depending on the material and the printer used, further phases may follow such as subsequent curing processes or, for bioprinting, a further incubation of the cells printed in the desired form in order to obtain functional tissue [6].

## 3. Bioprinting

To understand the significance of the following 3D printing processes for modern medicine and tissue engineering in particular, it is important to take a closer look at the functioning of so-called bioprinting itself.

The basic principle of bioprinting is to produce artificial tissue or, ideally, entire organs through the interaction of cells, growth factors, and biological supporting tissues, and withal to come as close as possible to the normal human body in composition and function [7]. This makes use of the possibility of printing the fabric layer by layer into a three-dimensional structure according to the desired or required properties [3]. A basic distinction is made between the principles of “bottom-up” and “top-down” printing.

The top-down method is the more commonly used printing approach. A scaffold, which is custom-made for the respective application, is subsequently loaded with the desired cells or proteins and functions as an artificial extracellular matrix of the tissue, supporting its structure and the supply of nutrients [8]. The scaffold itself can also be created using 3D printing techniques and loaded with growth factors such as Vascular endothelial growth factor (VEGF) or Epidermal Growth Factor (EGF) as required to enable better ingrowth and differentiation of the cells in the desired direction. The cell viability in this approach depends, among other things, on the quality and properties of the scaffold used. In particular, creating a suitable porous structure to allow the ingrowth of vessels to ensure vascularization still poses a challenge in the field of tissue engineering [9]. However, oxygenation and the supply of the required nutrients and growth factors also have a direct effect on cell growth and, conversely, limits the maximum possible cell density with which the scaffolds can be printed without the resulting competition for these factors, leading to increased cell death [8,10].

With the newer bottom-up principle, on the other hand, the desired cells or proteins are first transformed into smaller nano- or microscale structures, which are then assembled to form the desired macrostructures and grow together through self-assembly, manufacturing their own extracellular matrix [11]. Again, desired growth factors can be directly imprinted into the structures. A great advantage of this process is the possibility of controlling the formation of the final structure even better, since the built-in nanostructures can be directly arranged according to the required properties [11,12]. This functioning also makes it possible to introduce a much higher cell density into the tissue than with the conventional top-down method [8].

## 4. Bioinks

The large number of different 3D printing applications allows a fundamentally wide range of materials to be printed such as metals, ceramics, or cellulose. However, the use of so-called bioinks is of particular importance when it comes to soft tissue printing applications.

The term “bioink” is used inconsistently in the literature. However, it usually refers directly to cell-loaded “inks”, with and without additional carrier substances used for encapsulating the cells, taking over the functions of an extracellular matrix and, in particular, protecting the cells from the shear forces and stress generated during the printing process. All other cell-free materials used in soft tissue printing are predominantly referred to as “biomaterials” [13]. A large number of works deal in detail with the individual materials available [14,15,16,17,18,19,20]. Nevertheless, a brief overview is necessary for further understanding of tissue engineering. 

For a material to be considered for soft tissue printing, it must meet the basic mechanical, chemical, and biological requirements [21]. For example, the material must have the necessary viscosity, which always depends on the intended printing application, to be printable at all. It must also be possible to cure the materials during or after the printing process in such a way that a satisfactorily mechanical stability of the later structure can be guaranteed. It should be possible to chemically modify the materials to facilitate cell growth and differentiation. The closest possible proximity to normal human tissue is desirable. Of course, the materials should be biocompatible and not toxic to the imprinted cells or the surrounding tissue after implantation [14,21]. In the case of printed scaffolds, the materials must be additionally biodegradable in order to make room for the extracellular matrix formed by the growing cells during the dissolution process, without the degradation products resulting from this having a negative effect on ingrowth.

Based on these requirements, three larger groups of possible printing materials have emerged, in particular:

1. Hydrogel-based materials (e.g., hyaluronic acid, collagen, or decellularized extracellular matrix from foreign tissue) have the advantages of high biocompatibility and biodegradability. A large number of hydrogels can also be photocured under UV light and modified, for example, by introducing growth factors or proteins. These properties make them very suitable for scaffold printing or encapsulating cells to create bioinks [17].
2. The second group of materials, cell aggregates, or pellets and therefore the actual “bioinks”, have been used in the past, among other things, for the scaffold-free printing of tissue. Several successful experiments were carried out with the cells of Chinese hamster ovaries (CHO) [22], which were imprinted as cellular aggregates or as pre-treated cell pellets in collagen and showed minimal cell death rates after finishing of the printing process [23]. Yu et al. isolated primary chondrocytes from the kneecaps of cattle to produce approximately 8 cm long printable “tissue strands”. These were printed completely without additional scaffolding and formed a complete cartilage tissue after two weeks of incubation [24].
3. In the last subgroup of biomaterials, composite bioinks and the bioactive molecules already above-mentioned can be summarized.

Composite bioinks include various nanomaterials such as AuNPs or AgNPs incorporated into the printing material [25,26]. These nanoparticles enable, among other things, a strong mechanical stability of the tissue or, in the case of AgNPs, electrical conductivity, which is needed and has already been used for printing a bionic ear [26]. Furthermore, a large number of inorganic fillers are currently the subject of efforts to increase the properties of the biomaterials in terms of biocompatibility, mechanical strength, and bioactivity on the encapsulated or sowed cells and are explained in more detail in other papers [14,15,27,28]. For example, bioactive glasses such as SiO_2_, Na_2_O, or CaO can be added to the hydrogels to increase the bioactivity of the resulting bioink, as ions released from these glasses into the hydrogel showed bioactive and osteogenic properties in previous studies, making it a promising approach, especially in bone tissue engineering [29,30,31]. In other studies, the use of clay as a bioink additive led to the higher mechanical stability and rigidity of the printed scaffold, similar to that of trabecular bone [28,32]. Furthermore, it could be shown that clay fillers are capable of slowing down the degradation process of the hydrogels, making it possible to slow down the release of additionally added drugs or growth factors in a more controlled manner over a longer period of time, instead of a fast and uncontrollable “release burst” [32]. A similar principle is followed in the addition of graphene. With this approach, not only are the mechanical properties, electronic conductivity, and the biocompatibility of the bioinks increased, but it becomes possible to release the encapsulated cells in response to a stimulus, preferably near infrared light, which allows a more controlled tissue modeling [33,34]. In some studies, it was shown that the addition of such nanoparticles increased the viscosity of the bioink and thus the pressure required (e.g., in extrusion-based printing, but only the increased pressure has an influence on the cell viability of the print, not the presence of the nanoparticles themselves) [35].

Bioactive molecules are, as already mentioned, for example, VEGF or IGF, which enable the printed cells to differentiate into the desired tissue or simply have a positive effect on cell growth. These molecules can be added directly to the bioink or printed into the tissues in the shape of microspheres for the controlled release of the molecules. It is also possible, for example, to take and purify the patient’s own blood. The resulting platelet-rich plasma (PRP) is freed from the cell mass of the blood and contains the patient’s natural growth factors. The PRP can then be used as an additive for bioink production [36,37]. Small peptides may also be deposited into the biomaterial. These can both additionally stabilize the later construct, and due to their high water-binding capacity, serve as dehydration protection for the inserted cells during the printing process [38]. Specially engineered microbes such as M13 phages have also been investigated as possible bioink additives. Due to their integrin and calcium ion binding domains, these phages were able to enable better cross-linking in the alginate used as the biomaterial as well as better cell adhesion [39].

## 5. 3D Printing Applications

**Figure 2:**
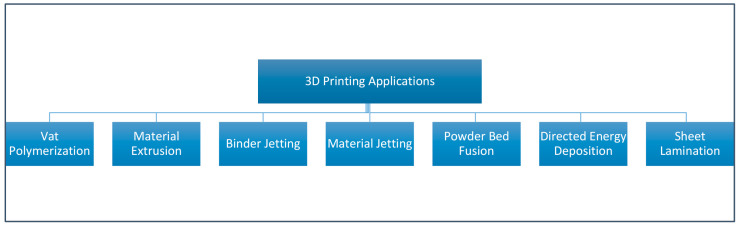
Overview of the various 3D printing applications, based on the subdivision of ISO 17296-2.

Since the introduction of the first 3D printing technology, a wide variety of printing processes with different applications and possibilities have been developed. The current ISO international standard 17296-2 distinguishes between a total of seven different printing processes: vat polymerization, material extrusion (ME), binder jetting (BJ), material jetting (MJ), powder bed fusion, directed energy deposition, and sheet lamination (Figure 2).

Each individual application is examined in more detail and, where possible, its previous use in tissue engineering will be discussed.

### 5.1. Vat Polymerization

The principle of vat polymerization is characterized by the curing of a photosensitive polymers using the light of specific wavelengths, mostly UV light. The resin used is therefore provided in a vat and cured layer by layer in the required areas, thus constructing the desired object piece by piece. The laser obtains the information for the resin points to be cured in the respective layer from the previously digitally determined coordinates. For this process to occur, a so-called photo-initiator must be added to the material. It absorbs the incident UV rays and develops active species, which ultimately ensure photopolymerization. Therefore, the parameters used for the laser source (e.g., wavelength, intensity and exposure time) have a significant influence on the printing process [40]. Furthermore, a large number of other factors have a direct influence on the quality of the printed object. In addition to the choice of resin, this also includes the aimed layer thickness or the post-curing time of the printed construct [41,42]. The advantages of these process types include relatively high resolution and short printing times. There is no need for a print head that could become clogged during printing and the material used is not exposed to shear stress, which could have a negative effect on the result [9,43,44]. In addition to stereolithography (SLA), the group of vat polymerization-based printing applications also includes the printing processes of two-photon polymerization (2PP), digital light processing (DLP), and continuous direct light processing (CDLP). The procedures differ from each other in a few essential points.

A further distinction is made between a top-down and a bottom-up approach. In the top-down process, the laser source is located above the vat and the construction platform moves down a defined length for each newly cured resin layer. In the bottom-up process, the laser source beams through the transparent bottom of the vat and the construction platform moves upward accordingly in the printing process [45,46]. Both methods have individual advantages and disadvantages. The top-down method seems to be more suitable for use in soft tissue printing. Due to the continuous upward pulling in the bottom-up process, forces act on the hardened material layers, which can be avoided in the top-down approach, thus the object rests on the construction platform during the printing process. As a result, this procedure has a gentler effect on the newly formed constructs. At the same time, the light source can hit the respective resin layer directly. In this way, disruptive factors on the laser energy such as scratching or soiling of the bottom of the vat as they can appear in the bottom-up method can also be avoided [45]. At the same time, the bottom-up variant is usually more cost-effective, as it needs less resin for printing and enables objects of smaller volumes to be printed [45,46].

A disadvantage of these printing applications is the need for support structures that have to be printed together with the actual object. These serve either to enable the printing of complex three-dimensional structures or to support overhanging structures during the printing process. The support structures have to be removed manually from the actual object after the printing process has been completed [40]. Furthermore, a big drawback of vat polymerization, especially for use in bioprinting, is the limited selection of suitable printing materials. Most of the resins used for photopolymerization form aggressive radicals under the influence of UV radiation and are mainly inappropriate for the medical sector.

Therefore, currently suitable resins are still quite limited. However, through constant research and further development, more and more biocompatible and photocurable materials are being created. The most prevalent resins in the medical field of application are multifunctional monomers made from methacrylates or acrylic esters with a low molecular weight (e.g., gelatin methacrylate (GelMA) or polyethylenglycol (PEG)) as they have the biocompatibility required for use in the human organism [42,45,46]. These materials come with the disadvantage that uncured resin residues can remain in the individual layers and thus negatively affect the mechanical stability of the structure [47]. The structures made from these materials also become mostly rigid and porous, which limits their possible application [42]. Therefore, the development of new, more flexible oligomers with better stability is the subject of current research as well as polymers with a so-called shape memory, being able to change their shapes under the influence of an external stimulus like heat or moisture, which could open up completely new possibilities for 3D printing. 

An overview of various examples for vat photopolymerization-based printing technologies in tissue engineering is summarized in Table 1.

#### 5.1.1. Stereolithography (SLA)

**Figure 3:**
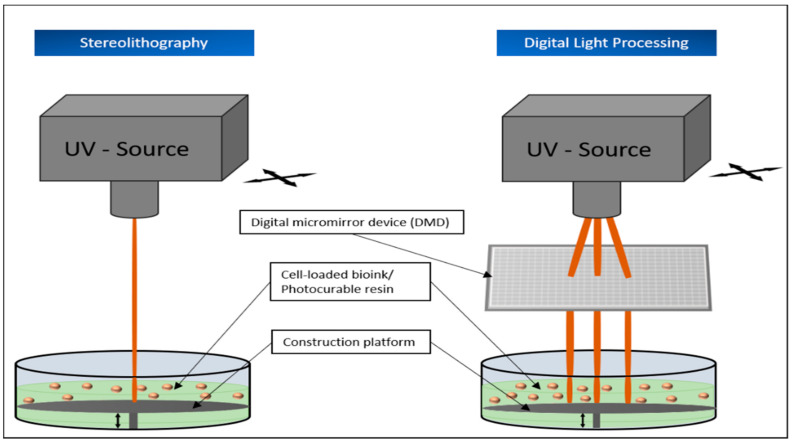
Schematic representation of stereolithography (SLA) and digital light processing (DPL) as top-down approaches. Both processes are based on crosslinking a photosensitive resin using a laser. SLA cures the individual points of a resin layer one after the other. Digital light processing can cure entire layers at once, using so-called digital micromirror devices.

The first 3D printing process ever used was the so-called stereolithography, developed by Charles Hull in 1984. This method uses a pool filled with light-sensitive liquid resin, a construction platform that can be moved up or down in the pool, and a UV laser for photocuring (Figure 3). In order to cure only the required points of the resin layer, either the laser itself or a motorized mirror is deflected according to the provided coordinates. Once all points of a layer are cured, the construction platform moves a defined distance to bring a fresh layer of liquid resin between the object and the laser. In this way, the desired object is put together layer by layer. After the printing process, the printed construction as a whole is cured again under UV light [42,44]. The quality of the object printed in this way depends not only on the selected materials, but also on the wavelength, energy, and spot size of the laser used for the curing process [42].

Stereolithography is used in the most diverse areas of medicine (e.g., to produce dental prostheses, surgical instruments or hearing aid components). In addition, the fields of application could be continuously expanded in the future with the development of further photocurable polymers. Among other things, the process offers the advantages of an enormous print resolution, which makes it possible to print individual layers with a minimum thickness of 0.025–0.050 mm and is considered to be one of the best resolution capabilities of any 3D printing application currently available [42,44].

Particularly in the field of tissue engineering, SLA is used for printing the scaffolds, constructions to usually imitate the extracellular matrix (ECM) of the printed tissue and enable the cells to adhere, proliferate, and also differentiate into the cell types desired for the respective tissue, depending on the epithelial growth factor, vascular endothelial growth factor, etc. added to the bioink [48,49].

Seck et al. used poly (D, L-lactide)-poly (ethylene glycol)-poly (D, L-lactide) (PDLLA-PEG-PDLLA) to produce a biocompatible and biodegradable resin. The scaffold made with this resin was then seeded with human mesenchymal stem cells, which showed satisfactory adhesion and proliferation on the printed scaffold after five days [50]. In another experiment, Grogan et al. used methacrylate gelatin (GelMA) for scaffold printing and sowed prepared human avascular zone meniscus cells onto it. After two weeks of cultivation, the resulting tissue was placed in an explanted model organ. Not only could it be shown that the GelMA used was not cytotoxic to the surrounding tissue, but also that the seeded cells were able to differentiate into the desired meniscus-like cells and integrate into the surrounding meniscus tissue, making this procedure a promising approach for the therapy of meniscus damage [51].

In the past, several studies on the printing of cells encapsulated directly in the material have been published. Encapsulated NIH 3T3 fibroblasts were placed directly into a mixture of PEG and GelMA and printed using SLA [52]. To avoid the potentially damaging effect of UV rays, a specially designed water filter was placed between the UV source and the resin. Immediately after printing, it could be shown that over 80% of the printed cells were still alive [52]. Using projection stereolithography, Lin et al. even achieved a cell survival of over 90% seven days after printing. The human adipose-derived stem cells (hADSCs) used in this experiment were placed in a mixture of polyethylene glycol diacrylate (PEGDA) and lithium phenyl-2,4,6-trimethylbenzoylphosphinate (LAP) as the photoinitiator [53].

#### 5.1.2. Two-Photon Polymerization (2PP)

Two-photon polymerization represents an improved type of stereolithography, as it clearly outperforms it in resolution and printing speed. The process is based on the principle of two-photon absorption, according to which a molecule is simultaneously excited with two photons of the same or similar frequency into a higher energy state [54]. Unlike the other vat polymerization processes, whose laser sources use light with wavelengths in the UV range (365–385 nm), 2PP uses light pulses in the near-infrared range (from 780 nm) to trigger photo polymerization [40,55]. A major advantage of this method is, among other things, that this reaction only takes place exactly at the focal point of the incident laser. This allows the laser to penetrate through several layers of the light-sensitive material without causing them to cure directly [56]. In this way, the entire printing process becomes much more precise and faster. Two-photon polymerization enables resolutions in the range of nanometers to a few micrometers [57,58]. A disadvantage is that this printing technique is still very expensive and therefore not necessarily affordable for everyone. The above-mentioned advantages of 2PP have been used in the past by Ovsianikov et al., among others, in various experiments to print scaffolds with the desired structural properties and to test the materials used for their biocompatibility [59,60,61]. For example, methacrylamide-modified gelatin (GelMod) was used for scaffold printing and seeded with porcine mesenchymal stem cells, showing sufficient adhesion and proliferation [59].

#### 5.1.3. Digital Light Processing (DLP)

DLP was developed in the 1980s by Texas Instruments. In contrast to SLA, DLP does not cure individual points of a resin layer one after the other, but all relevant areas of the respective layer directly, using a digital light projector, which makes the whole process much faster than SLA (Figure 3). To make this possible, the DLP method relies on digital micro mirror devices (DMD). These usually consist of 1–2 million of the smallest controllable mirrors, arranged only a few micrometers from each other and can be aligned in such a way that the incident laser light is deflected onto the photosensitive resin according to the desired areas of a layer. Based on the digitally calculated data, this so-called dynamic mask is redefined for each 2-dimensional layer of the object and aligned by the DMD [62,63].

Following this principle, the speed of this printing process mainly depends on the thickness of the individual layers [3]. Furthermore, DLP also has a very high resolution, which can reach up to six micrometers, depending on the material used for printing [63].

In the past, this fast and tissue-friendly process has already been used in different approaches for tissue printing. For example, model tissues for the simulation of lungs or liver [64] have been created with the help of DLP.

CDLP, on the other hand, differs from DLP primarily in the fact that the design platform moves continuously during the printing process, thus accelerating the entire process even further [65].

### 5.2. Material Extrusion (ME)

Although there are various subtypes of extrusion-based printing, the basic principle remains the same. A continuous stream of the material to be used is pressed by a mechanical or pneumatic force from a moving print head onto a corresponding construction plate until the desired object has been completed in this way [5].

**Figure 4:**
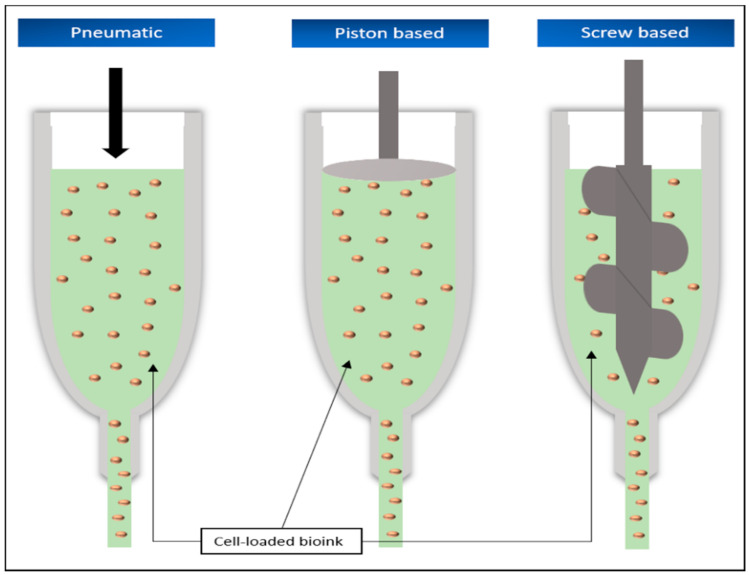
Schematic representation of the extrusion-based printing technologies. All applications have in common the use of a force directed at the nozzle to eject the material.

The subtypes differ primarily in the way the material is pressed out of the nozzle, which moves according to the digitally created or scanned model of the object. The classification is mainly defined into pneumatic and mechanical applications, the latter being further divided into piston-based and screw-based systems (Figure 4). A variant of extrusion-based printing is the fused filament fabrication (FFF).

In the pneumatic extrusion application, this process is achieved with the help of compressed air, which is fed into the print head loaded with printing material by means of an air pump. The force applied depends on the strength of the pressure built up. Particularly for working with bioinks for tissue engineering, it is important that the air used is sterilized before contact with the material to avoid contamination [9,66].

The principle of piston-based printing relies on, as the name suggests, the force of a piston acting directly vertically on the printing substance, pushing the material out of the nozzle. Therefore, the piston used is usually connected to a motor via a guide screw [5,9].

The screw-based method, on the other hand, dispenses without an additional piston and directly uses the rotational movement of the screw, which is generated by connection to a motor, to push the material from the nozzle [5,9]. Several factors have a direct influence on the result and the speed of these printing processes and can be directly modified. For example, the diameter of the print head, the speed of material-ejection, the temperature increase that occurs during the printing process, or the viscosity of the material can have an immediate influence on the result of the print [9,67].

Fused filament fabrication can be described as a subset of extrusion-based techniques. In this process, the polymers used are heated either in the cartridge or through the print head and forced out of the nozzle via a feeding mechanism like the aforementioned techniques. This printing technique is particularly characterized by its ability to print complex geometric shapes with great precision. These properties make this approach particularly attractive for the production of scaffolds as the basis for soft and hard tissue models [67].

Extrusion-based 3D printing systems are among the most widely used 3D printing applications. Particularly in the field of bioprinting, this technology is characterized by a high printing speed, which makes it possible to print even larger tissue constructs in a relatively short time [68]. In addition, the extrusion-based printers currently available are low in purchase, especially when compared to other current 3D printing technologies. The structure and function of these printers are easy to understand and therefore very user-friendly, especially for beginners, without the need for previous extensive induction [69]. At the same time, extrusion-based printers, in addition to stereolithography, are best suited for printing porous lead structures such as those required for creating sufficient vascularization of artificial tissue [68]. Furthermore, these printers dispose of a wide range of usable biomaterials. In addition to cell-loaded aggregates, this also includes hydrogels with and without cell-loading, micro-carriers, or decellularized matrix components [67].

Despite these advantages, the use of extrusion printers is accompanied by a number of restrictions that must be observed. With a minimum possible print resolution of 50 µm, controlled placement of cells in the tissue structure is only possible to a limited extent [69,70]. The quality of the print is also significantly influenced by the choice of biomaterial as it must have a sufficiently low viscosity in order to avoid clogging the nozzle during the printing process. At the same time, the viscosity must be high enough so that no droplets form when the nozzle emerges, but the material can be applied continuously [71]. Particularly in the case of bioinks with a high cell density, strong shear forces can appear in the nozzle tip, having a negative effect on cell viability [67,70]. Given the necessity of taking these limitations and requirements associated with extrusion-based printers into account, choosing the right biomaterial becomes more difficult. Several papers have therefore explicitly dealt with the question of potential biomaterials and their advantages and disadvantages for this printing approach [71,72,73,74,75].

In all of its subtypes, this printing application is one of the most widespread methods in the field of bioprinting and has also been applied frequently in the field of soft tissue printing (Table 2).

Yeo et al. used a modified extrusion printer with a core-sheath nozzle to print osteoblast-like (MG63) cells and human adipose stem cell (hASC)-loaded collagen surrounded by a protective layer of pure alginate in a 3-dimensional porous mesh structure. Using an aerosol process, calcium chloride solution was added during the printing process to crosslink the bioink and alginate and ensure that the cell-bearing bioink remained at the center of the alginate coating. One day after printing, cell viability of approx. 90% was observed with correspondingly good metabolic activity [76].

A particularly interesting work is by Kim et al. from 2017, who combined extrusion-based printing and inkjet-based printing to create a skin model consisting of dermis and epidermis. Using an extrusion-based printer, a scaffold of polycaprolactone (PCL) and 25% gelatin hydrogel was printed to create a transwell system within the tissue and was additionally printed with 2% human primary dermal fibroblasts (HDF) populated collagen. This step was repeated several times up to the desired height of the construct of 3.5 mm. Subsequently, inkjet-based printing was used to apply human epidermal keratinocytes (HEKs) to the resulting construct. After 14 days of cultivation, it could be seen that the printed fibroblasts exhibited stretching behavior very similar to normal human skin. The keratinocytes developed an epidermis-like stratification. The production of collagen 1 and dermis-specific differentiation markers in the artificial skin were also demonstrated [77].

### 5.3. Binder Jetting (BJ)

**Figure 5:**
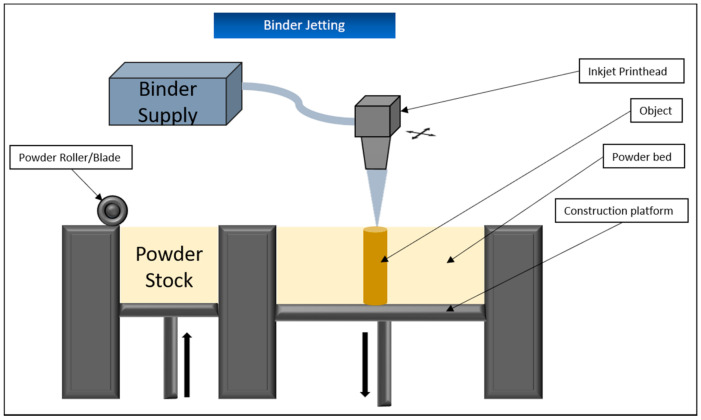
Schematic representation of the binder jetting printing technology. Powder layers that are added continuously are hardened at the required points using a liquid binding agent.

Binder jetting is based on the interaction of two different components. The actual printing material is in powder form on a vertically movable platform. A print head applies a liquid binder to the top layer of powder at the selectively required areas and thus causes it to harden. The platform is then lowered a little and fresh powder is spread over the previously hardened surface, usually using a blade or roller (Figure 5). The surrounding non-hardened powder material serves to stabilize the structure, so that no additional scaffolding is required in this procedure. Once the object has been completed in this way, resident and unneeded powder is removed via compressed air and the entire structure is cured by sintering or the addition of a polymerizing resin [83]. This process usually takes longer than the actual printing process.

One advantage of this process is that by using different print heads with different fixing materials, various colors and properties can be printed into the object. In addition, binder jetting offers a high resolution of 80–250 micrometers as well as the possibility to use a very wide range of materials, especially different metals, as long as they are in powder form, for printing [84].

A disadvantage of this method is especially the mechanical stability of the objects. Despite the subsequent curing process, a varying degree of porosity within the printed material cannot be completely avoided, which means that objects constructed by binder jetting are usually not printed for purposes subject to high mechanical stress, but for surgical planning [85]. In addition, the curing processes used generally lead to the fact that the items are rather roughly organized in their structure and shrink during the process, which is very difficult to control in advance and further limits their areas of application [86]. While binder jetting is already used in the food industry and in the processing of pharmaceuticals, it has received little or no attention in the production of soft tissue for the reasons above-mentioned.

### 5.4. Material Jetting (MJ)

Material jetting is most similar to the well-known inkjet printers, since in both processes, the respective material is sprayed on at the required points via the print head. As in all 3D printing processes, this takes place in many individual layers one after the other. Normally, the print head is freely movable in the horizontal plane and has several individual small glands that can be switched on or off as required. Material jetting does not use simple inks, but mostly liquid photopolymers or synthetic resins, which are cured by subsequent UV treatment, similar to the SLA, or waxes, which are first heated and sprayed in liquid form and then cured while cooling. As usual, the design platform is lowered a little after each layer to allow the next one to be printed [85]. By using multiple print heads, materials of different colors and properties can be printed into the construct [83,85]. In order to ensure sufficient stability of the construction during the printing process, a supporting substance is usually sprayed on in addition to the actual object via a further print head. These structures must be broken off from the actual object after printing [87]. Although this process offers a high printing speed, it is important to remember that the material used must have a low viscosity to be sprayed through the nozzles of the printer [83].

Just like stereolithography and material extrusion, material jetting is suitable for printing living cells [21,43,88]. Therefore, hydrogels such as chitosan or collagen are the materials of choice to encapsulate the cells and protect them from the forces that occur in the printing process [66,89,90].

Nevertheless, this application is also associated with a number of disadvantages that must be taken into account when used for classical bioprinting.

As already mentioned, the biomaterials used must have a sufficiently low viscosity in order to avoid clogging of the nozzles. Consequently, the maximum possible cell density of the bioinks used is also limited, as a higher cell density thickens the whole bioink [66,83,89]. The storage time of the material in the printer’s cartridge also affects the printing process. At first, the loaded cells are evenly distributed in the bioink, but eventually will settle over time, resulting in a concentration and viscosity gradient that can ultimately lead to clogging of the nozzles even when using bioink with an initially sufficient cell density. This is called the settling effect and must be addressed when choosing material jetting for printing [91,92]. At the same time, this approach is also characterized by a very good resolution of up to 10 µm with a drop volume of 20 picolitres, which means that very precise structures can be printed [89].

Aside from its usage in bioprinting, material jetting is often used for prototypes of later projects or, in medicine, for the production of anatomical models because of the ability to print different materials of different colors into one construction. The latter can be used, for example, in patient education of disease concepts or for practice before surgical interventions [93].

**Figure 6:**
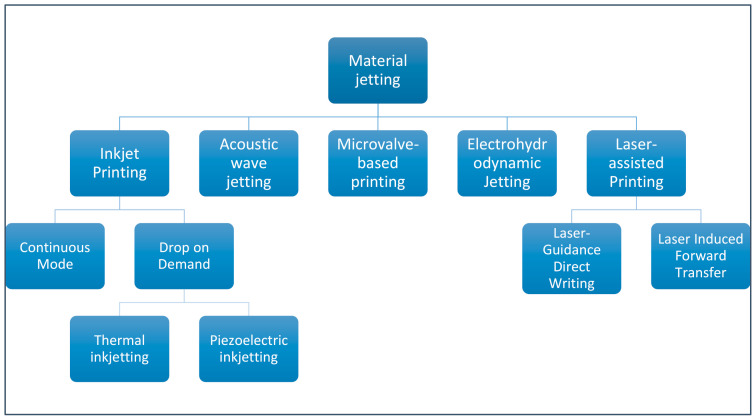
Overview of the different subtypes of material jetting.

Generally, the basic principle of MJ can be divided into different subtypes (Figure 6). The five main types are inkjet printing (IP), acoustic wave jetting (AWJ), microvalve-based printing (MBP), electrohydrodynamic jetting (EHDJ), and laser-assisted printing (LAP). LAP is a little different from the other subtypes and includes laser induced forward transfer (LIFT) and laser guided direct writing (LGDW). Inkjet printing is further divided into the continuous stream (CS) mode and the drop on demand (DOD) system, where material drops are generated individually and sprayed only when needed [94,95]. DOD is further divided into thermal and piezoelectric inkjetting.

A summary of the large number of studies carried out by material jetting-based 3D printing technologies in the field of tissue engineering is given in Table 3.

#### 5.4.1. Continuous Mode (CS)

**Figure 7:**
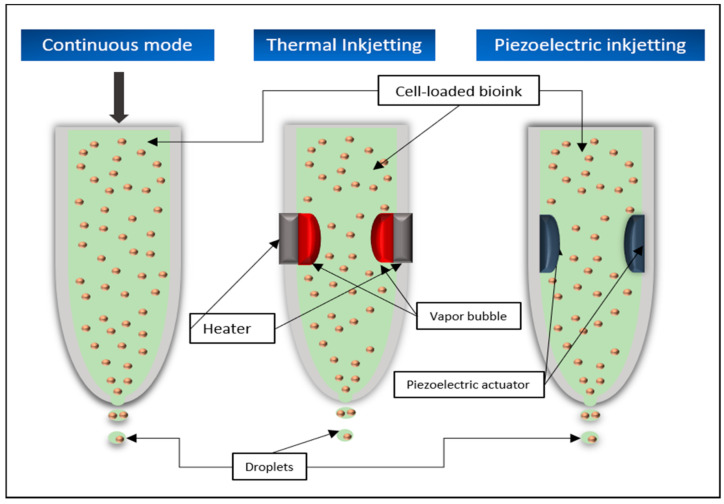
Schematic representation of various material jetting-based printing technologies. All processes rely on the printing material being sprayed drop by drop from a nozzle.

In continuous mode, the liquid material is sprayed out of the nozzle by a constant pressure and splits into individual drops after leaving the spray head (Figure 7). The time of splitting can be influenced, for example, by vibrations, to make sure that the jet breaks up into individual drops of continuous size. To ensure that the material drops are only sprayed onto the structure at the desired point, they must be deflected in the required direction after leaving the nozzle. Therefore, the drops either have an electric charge of their own or first pass through a charged field and in turn receive such charge. The particles are then deflected to the desired position in a deflection field.

CS can generate droplets much faster than the DOD method, but the latter offers the advantage that much smaller droplets and thus a better resolution of the later object are possible. There is also the problem that unused drops must either be removed from the system or returned to it, which may result in a waste of material or the recycled substance may no longer be sterile [9,94].

#### 5.4.2. Thermal Inkjetting

Thermal inkjetting uses a thermal actuator that is heated by an electrical impulse for a very short period of time to temperatures of 200–300 degrees Celsius. The resulting evaporation processes lead to the formation of a bubble in the material container. This bubble becomes larger and eventually explodes, exerting pressure on the material already in the nozzle and thus helps to overcome the surface tension prevailing there, causing the desired drops to leave the spray head (Figure 7). The time for the trigger to heat up is usually too short to damage the material, which means that even living cells can be printed [5,9,66].

Thermal inkjetting was used by De Coppi et al. to embed amniotic fluid-derived stem (AFS) cells into a scaffold of collagen/alginates. The resulting construct was cultured for one week in an osteogenic inducing medium and subsequently implanted into immunodeficient mice. After eight weeks, the cell-printed scaffold was found to have formed highly mineralized tissue. A micro-CT scan after 18 weeks showed that the construct had formed a bone-like tissue with even greater density than that of a mouse femur [96].

The biocompatibility of living mammalian cells with thermal printing was also investigated in studies by Xu et al., where Chinese hamster ovaries (CHO) and embryonic rat motoneurons were printed on a “biopaper” consisting of soy agar and collagen hydrogels. Subsequent evaluations showed that less than 10% of the CHO cells had died during the printing process [97].

#### 5.4.3. Piezoelectric Printing

Piezoelectric printing works on a very similar principle. Here, a piezoelectric actuator is made to expand or contract in dependence of an applied alternating voltage. This rapidly changing deformation leads to the formation of pressure waves within the printing material, which provide the necessary energy to overcome the surface tension in the nozzle [9,43] (Figure 7). By modulating the frequency and extent of the deformation of the actuator, the size of the emerging drops can be influenced [94].

In 2014, Xu et al. used a bioink, consisting of fibroblasts and sodium alginate and demonstrated that cell density of the bioink was inversely proportional to drop size and speed of the printing process [98].

#### 5.4.4. Acoustic Wave Jetting

**Figure 8:**
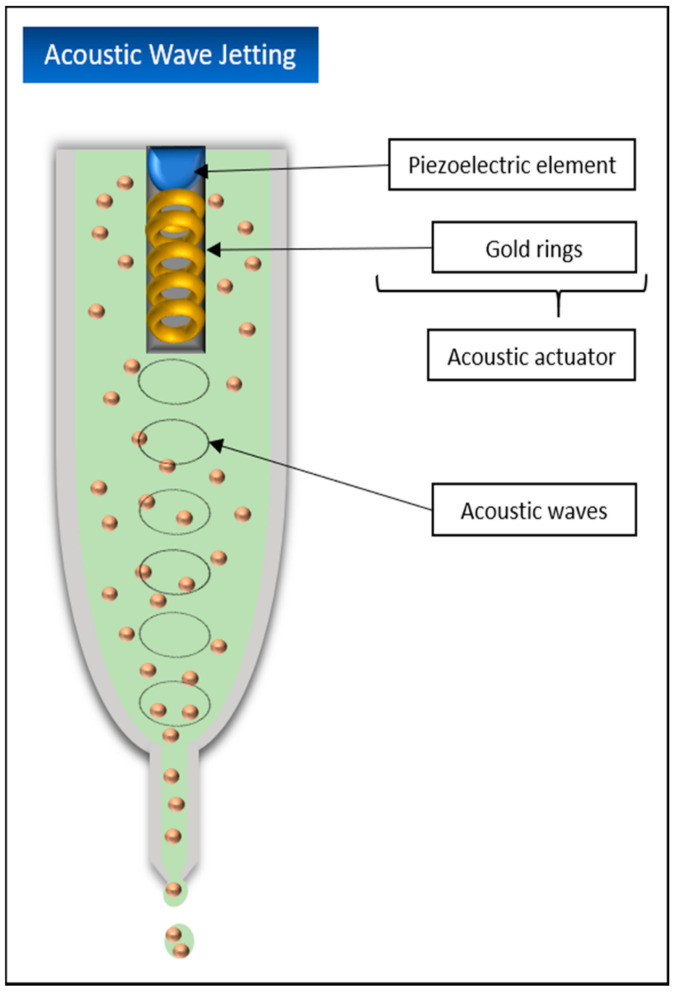
Schematic representation of the acoustic wave jetting. The generation of sound waves is used to spray the print material out of the nozzle.

Acoustic wave jetting is a comparatively new printing process and is very similar to inkjet printing methods in its functionality. However, the printing material is not located in a nozzle but in an open pool with the acoustic actuator located in its center and with the nozzle at the end. The actuator consists of a piezoelectric element and small interconnected gold rings. When an electric current is applied, this actuator emits ring-shaped acoustic waves that are vectored directly at the nozzle tip, thus overcoming the surface tension and causing the material to be sprayed out in drop form [43] (Figure 8).

Promising results with acoustic wave jetting were obtained by Demirci et al. in experiments with mouse embryonic stem cells, fibroblasts, or AML-12 hepatocytes encapsulated in various biological fluids such as agarose hydrogels. Not only could cell viabilities of more than 89.9% be shown, but also the possibility of this application to specifically move only single cells or RNA/DNA of single cells in the picolitre range, which could make this method very important for different research fields in the future [99].

#### 5.4.5. Electrohydrodynamic Jetting (EHD)

**Figure 9:**
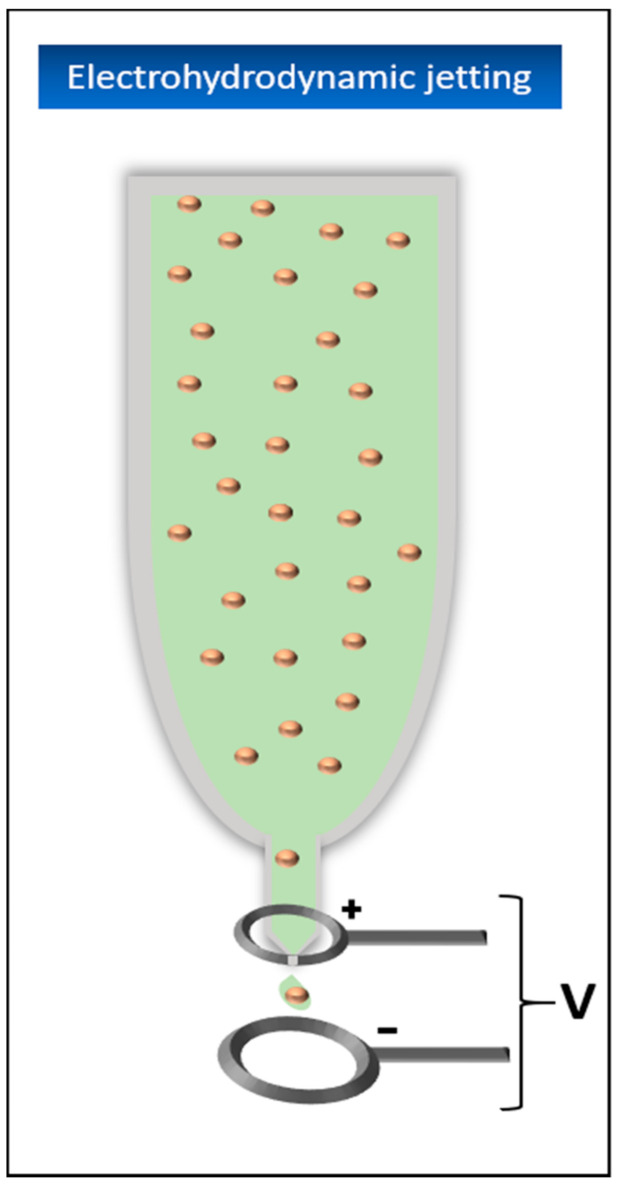
Schematic representation of the electrodynamic jetting. The printing material is sprayed out of the nozzle with the application of an electric field.

In contrast to the material jetting processes described so far, in electrohydrodynamic jetting, the material is not forced out of the spray head, but rather pulled out by electrodynamic forces. For this purpose, an electrical field is established between the nozzle and the design platform (Figure 9). This ensures that moving ions migrate to the material meniscus at the tip of the nozzle. The comb forces acting there between the ions cause the meniscus to transform into a conical shape, the so-called Taylor cone. If the resulting electrostatic forces are strong enough, the surface tension of the material is overcome and a jet or single drops are pulled through the electric field [100]. By changing the electric field, the drop size can be influenced. A higher voltage leads to smaller drops [101].

The nozzle itself usually consists of a glass capillary, which is often additionally coated with a thin metal layer to avoid adhesion and clogging of the nozzle by the material. It is continuously supplied with new material by a syringe pump or a pneumatic pressure system [102,103].

The material jetting processes described so far have the disadvantage that the diameter of the nozzle must be as small as possible to generate correspondingly small drops. However, this diameter is limited to a certain minimum because the pressure building up in the spray head should not become too large and the material should not be damaged when it exits through the nozzle. Accordingly, the viscosity of the material must be as low as possible to be able to print at all. Exceedingly small nozzle diameters also tend to clog up during printing, disadvantages that are particularly noticeable when printing with living cells. With EHD jetting, the diameter of the material droplets is independent of the diameter of the nozzle. Even with a diameter of more than 100 mm, drops of only a few micrometers in size can be produced, which avoids the problems above-mentioned, allowing the printing of thicker materials and improving the resolution of the process enormously [104].

#### 5.4.6. Microvalve-Based Printing

**Figure 10:**
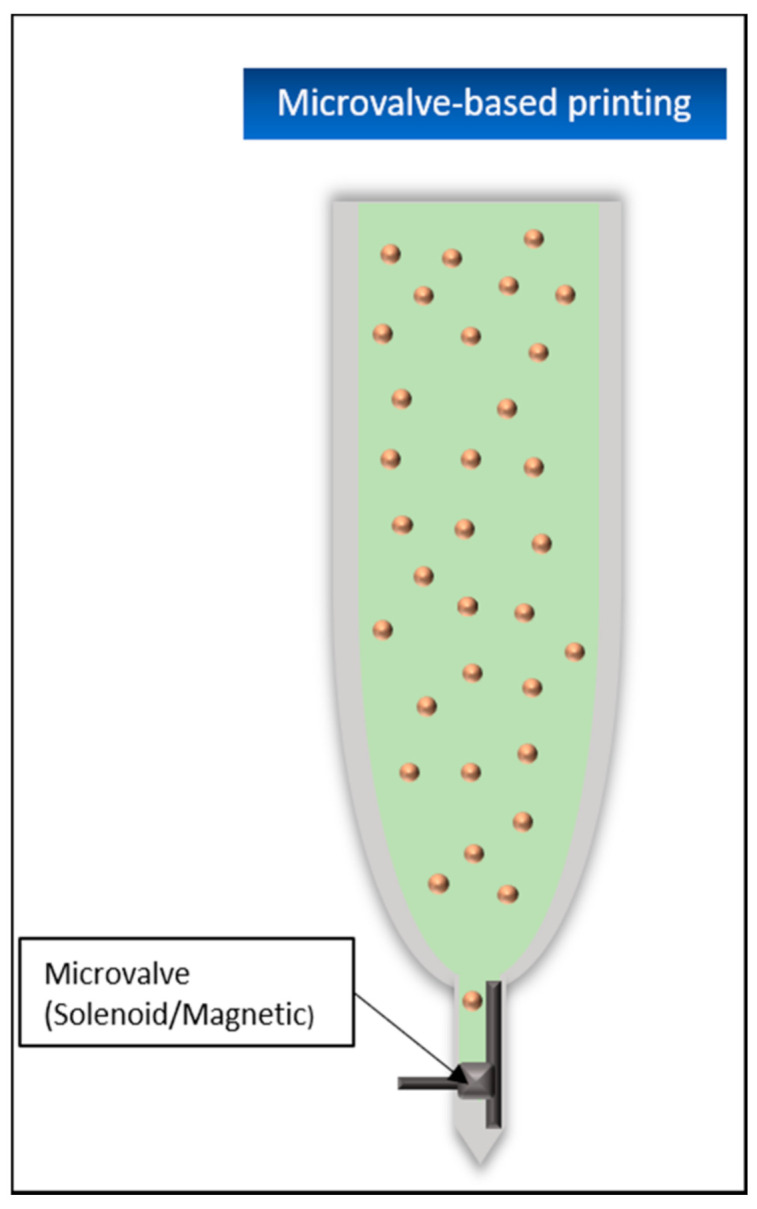
Schematic representation of the microvalve-based printing. The dispensing of the drop-shaped material is controlled by the combination of a magnetized coil and a small plug on the nozzle tip.

This process uses electromechanical or solenoid valves to precisely control the outflow of materials from the nozzle. These microvalves usually consist of a solenoid coil and a plug closing the nozzle (Figure 10). When an electric current is applied, the coil generates a magnetic field that pulls the plug out of the nozzle opening. The printing material is pressed into the nozzle by a constant pneumatic pressure. If the nozzle is opened and the applied pressure exceeds the surface tension of the material at the nozzle opening, it will be ejected. By varying the opening time of the nozzle and the applied pressure, this process allows either continuous or DOD printing [105]. Usually, this method uses several print heads with partially different materials at the same time, which is one of the big advantages of this method, because, especially in bioprinting, different cell types can be printed into the same tissue [106]. Another advantage is a relatively high printing rate of about 1000 drops per second and, for bioprinting, a cell viability of about 86%. However, as with almost all nozzle-based printing techniques, a major disadvantage is the tendency of the nozzles to clog during the process, which limits the maximum possible viscosity of the materials used and the bioinks in their possible cell concentration [43,106].

Several soft tissue printing trials have been conducted in the past using microvalve based printing. In 2009, Lee et al. used this printing application to create a tissue consisting of fibroblasts and keratinocytes with the goal of mimicking the multi-layered construct of human skin. First, collagen was applied to a surface coated with sodium bicarbonate (NaHCO3), which acted as a crosslinking agent. The prepared cells were imprinted into this hydrogel layer, which was thus partially stimulated to crosslink, and a further layer of NaHCO3 was applied afterward to stimulate the hydrogel surrounding the cells to crosslink, and at the same time, act as a basis for repeating the previous steps. The fibroblasts were embedded in the second printed collagen layer. After six cell-free collagen layers in between, the keratinocytes were embedded and covered again by two additional collagen layers. To test whether the soft tissue could be printed directly into an uneven surface, an artificial wound was created using polymethylsiloxane (PDMS) and printed according to the procedure described above. With viabilities of the printed cells (approx. 95% FBs and 85.5% for KCs) in direct comparison to manually applied cells (approx. 96.6% control FBs and 83.9% control KCs), no significant influence of the printing application on the survival of the cells was shown. Cells directly imprinted into the wound model also showed a satisfactory proliferation rate afterward [78].

Quite similar experiments were conducted in 2013. Here, three layers of fibroblasts were printed first, always with two layers of pure collagen below and above the FBs. Then, two layers of keratinocytes were printed on the top collagen layer. NaHCO3 was again used as the crosslinker for the collagen layers. The resulting construct was cultivated for 4–8 days and then exposed to the air–liquid interface (ALI) for another 10–14 days to stimulate the keratinocytes to proliferate and form the desired stratum corneum. With this procedure, cell viabilities of both cell types of approx. 98% could be achieved. It was also shown that the printed skin model was much more stable and less susceptible to shrinkage during cultivation compared to manually produced tissue [6].

In 2018, MBP was also used to produce a pigmented human skin-like artificial tissue. For this purpose, melanocytes (MCs) were added into the construct in addition to FBs and KCs. First, the collagen-fibroblast matrices were printed in 6-well culture inserts and cultured for four days. KCs and MCs were then printed directly onto the fibroblast collagen layer, with one MC drop directly surrounded by eight KC drops. The tissue was cultivated for seven days and then stimulated at the ALI for further maturation for another four weeks. In comparison to a tissue produced manually in the “classical way”, the advantage of the bioprinting approach was, among other things, that the microstructure of the tissue could be much better controlled and this way, the porous structure required for good cell–cell interaction could be constructed in the collagen–fibroblast matrix. In addition, the printed tissue showed a uniform pigmentation, in contrast to the manual approach, which showed a central pigmented area surrounded by unpigmented parts. The desired stratification of the KCs up to their differentiation into corneocytes on the surface of the tissue could also be observed, thus demonstrating the basic possibility of producing pigmented artificial skin tissue [107].

#### 5.4.7. Laser-Assisted Printing (LAP)

##### Laser-Induced Forward Transfer (LIFT)

Laser-induced forward transfer was initially developed for the processing of metals but has since then found its way into bioprinting in particular. This technique can now be used to print different hydrogels with or without encapsulated cells.

**Figure 11:**
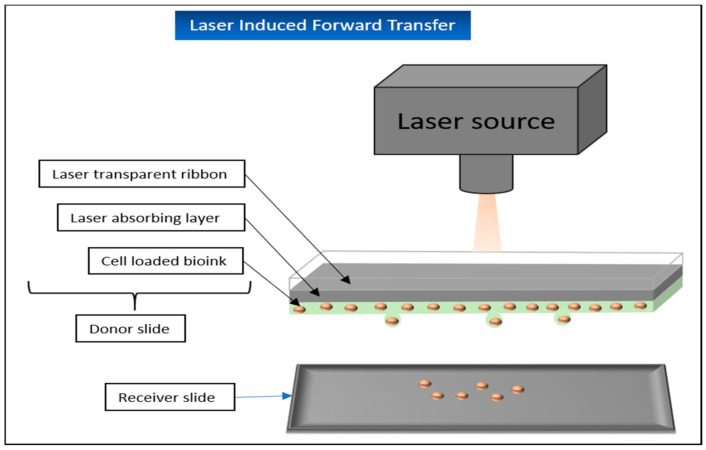
Schematic representation of the laser induced forward transfer. Printing material is located on a specially prepared “donor slide” and is sprayed onto a “receiver slide” in droplet form with under usage of a laser source.

The essential elements for laser-induced forward transfer are a pulsed laser, a focus-system, a “donor-slide”, and the opposite “receiver-slide” [88]. The donor-slide consists of a ribbon, usually made of transparent glass, coated with a laser-absorbing layer, mostly made of metal and a thin layer of the printing-material additional on one side on top of the absorbing layer. When the beam of the pulsed laser hits the donor-slide, the metal layer is vaporized by the incident energy. As a result, drops are released from the printing material and fall onto the receiver-slide [21,66] (Figure 11).

LIFT has several clear advantages to offer. Since no nozzle is required, the shear forces normally acting on the material are also eliminated, which ensures greater cell viabilities for use in bioprinting [89]. At the same time, this eliminates the problem of nozzle clogging and also allows materials of higher viscosity to be printed without any problems.

Disadvantages of this process are, among other things, the usually complicated preparation of the donor slides, the currently still relatively high costs of the process, and the contamination of the printed tissue with metal residues from the absorption layer [21].

The benefits of LIFT for soft tissue engineering have been demonstrated repeatedly in the past. Michael et al. used this technique to print and implant skin-like tissue in vivo. Twenty layers each consisting of collagen loaded with NIH3T3 fibroblasts and 20 layers of collagen loaded with HaCat keratinocytes were printed on a Matriderm^®^ membrane as a stabilization matrix. After one night in the incubator, the construct was implanted in vivo into prepared mouse skin fold chambers where the printed keratinocytes showed the characteristic stratification of the epidermis, although slightly thinner than in normal mouse skin. In the meantime, the fibroblasts occasionally grew into the Matriderm^®^, showing the tissue’s ability to integrate into the existing wound. After 11 days, the artificial tissue had grown together with the surrounding tissue. Small blood vessels could also be detected, which grew from the original wound bed into the printed tissue [108].

Koch et al. used a LIFT technique to print NIH3T3 FBs, HaCat KTs, and human mesenchymal stem cells (hMSC) and to examine cell viability, proliferation, and apoptosis rates. Potential DNA damage caused by printing was also a focus of the study. Gold served as the energy-absorbing coating in this experiment. The cells remaining on the donor slide after the printing process were considered as the control group. The local transfer efficiency (i.e., the percentage of cells that reached the recipient plate by laser transfer) reached over 90%. For the FBs and KCs, a cell survival of about 98% was determined immediately after the printing process was completed. The survival of the hMSCs was over 90%. Proliferation of the cells was assessed both by hemocytometer and by measurement of metabolic activity. In both cases, a continuous proliferation of the cells could be detected since day 1. Neither cell survival nor proliferation showed significant differences to the control group. Using the comet assay method, it was demonstrated that the LIFT process did not cause a significant increase in DNA damage in the printed cells. Of particular interest was also the finding that the printed stem cells retained their typical surface markers (CD44, CD105, CD29, and CD90) even after the experiment, demonstrating that no change in the immunophenotype of the stem cells was triggered by the printing process. This could open up further application possibilities of bioprinting in the future, in which printed stem cells can be imprinted into the injured tissue and only differentiate into the respective source tissue [109].

##### Laser-Guidance Direct Writing (LGDW)

**Figure 12:**
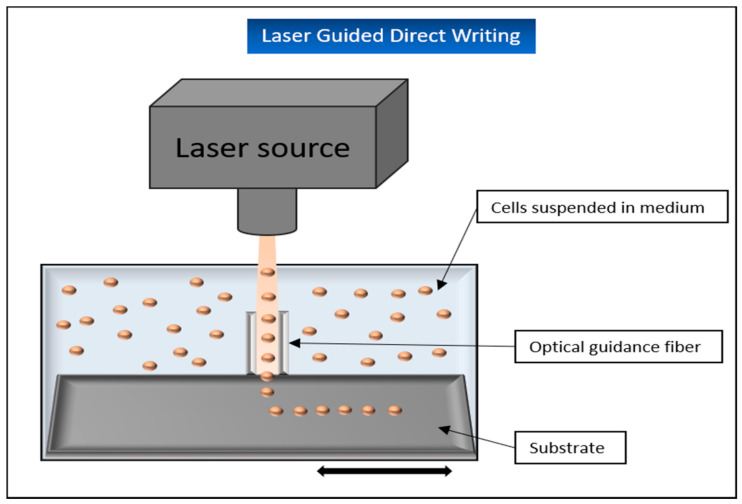
Schematic representation of laser guided direct writing. Cells are directed from a liquid solution onto the substrate using a weak laser beam.

An interesting but currently little used and investigated laser-based printing technology is laser-guidance direct writing. This method takes advantage of the fact that by using a weak laser beam that hits particles in a liquid solution, a gradient force can be built up to “trap” and move them. These particles are simultaneously pulled into the center of the beam and pushed in the axial direction of it [9,110] (Figure 12).

For this procedure to work, it is important that the refractive index of the deflected particle is greater than that of its surrounding medium. If, for example, living cells are used in a suspension, they can be positioned on a target surface using the power of a weak laser. If this surface is moved relative to the light beam, these cells can even be placed on the surface in desired patterns [110]. To further optimize this process, hollow optical fibers can be integrated into the design. These offer the advantage that the convective fluid forces prevailing in the suspension do not influence the optical forces of the light beam. The hollow fibers also allow the laser to be directed up to several centimeters. In this way, the particles or cells can be transported over longer distances. The advantages of this method are quite obvious: the cells can be placed directly and completely without any further chemicals such as crosslinkers and with an accuracy of less than 1 mm [110].

A disadvantage of this method is that although 100–1000 cells can be printed very precisely, this process takes several hours, whereas other bioprinting technologies can print larger areas much faster [111].

A promising attempt was made in the past by Odde et al., when embryonic chicken spinal cord cells were deflected onto a glass surface using a laser (approx. 109 W/m^2^ at 800 nm), which was directed through an optical lens with a low aperture and a hollow optical fiber. This experiment demonstrated that the cells can be precisely placed via LGDW, and additionally, that the cells remain viable after this procedure [111]. The same research group used the technique to place human umbilical vein endothelial cells (HUVEC) on Matrigel, as an angiogenesis assay system, to stimulate differentiation into a vascular system. By adding 40,000 cells/mL rat hepatocytes to the applied HUVECs, it could be shown after a few days that the printed endothelial cells were able to form a vascular scaffold for the hepatocytes and thus promote the formation of sinusoid-like structures in vitro. The resulting structures showed CYP450 gene expression and activity as well as continuous expression and secretion of albumin for the subsequent 2-month observation period [112].

### 5.5. Powder Bed Fusion/Selective Laser Melting (SLM) and Selective Laser Sintering (SLS)

While the terms “selective laser melting” and “powder bed fusion” can be used synonymously for the same process, there are some significant differences to selective laser sintering. For simplicity, both processes are explained together in this chapter.

The term “melting” refers to the complete heating of the powdered material above its melting point, whereas “sintering” only heats the material surface. As a result, sintered materials have a more porous structure and a rougher surface than fully melted materials, which have a correspondingly higher density and thus better mechanical stability. This comes with the advantage that objects produced by SLM, unlike SLS, do not usually require any additional curing processes afterward [121].

However, when selecting the materials for SLM, it is important to ensure that they do not differ too much in laser absorption or surface tension, in order to avoid structural weaknesses in the object later on. This limitation results in fewer materials being available compared to SLS [122].

As for both applications, the tank containing the powdery printing material is first heated to temperatures just below the required melting point of the respective substance. Due to this preliminary step, the laser later needs less energy to reach the required melting point and larger temperature fluctuations within the substrate are avoided, which has a positive effect on the structural quality of the later object [85].

**Figure 13:**
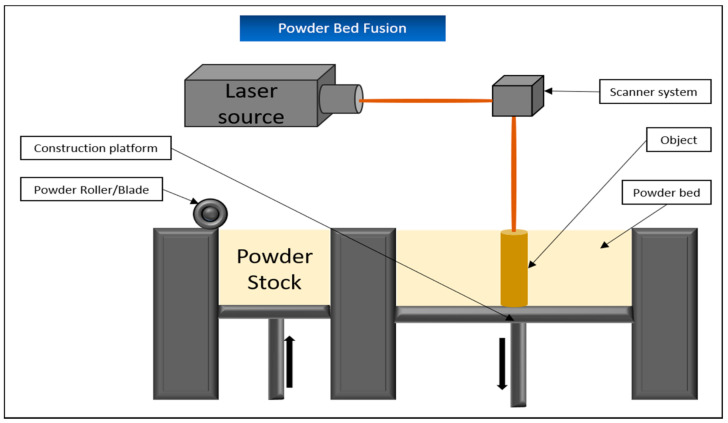
Schematic representation of the powder bed fusion. The continuously applied layers of the material powder are hardened at the required points using a laser source.

Then, similar to binder jetting, a blade applies a thin layer of powder to the construction surface. The powder is heated by the laser at the required points until it melts (or sinters). Afterward, the construction surface is lowered by layer thickness and the next layer of powder is applied (Figure 13). The powder that is not cured serves as a supporting structure in this process and can be removed mechanically afterward [87].

SLS is very well suited for the production of various scaffolds for bioprinting, since their porous structure, which is important for cell growth and tissue development, can be very well controlled. In the past, for example, the process has been used to produce collagen and gelatin-coated scaffolds from polycaprolactone (PCL) and has shown good results in the production of artificial cartilage tissue [123] as well as in the proliferation and osteogenesis behavior of subsequently added ASCs [124].

In vivo experiments with scaffolds produced via SLS demonstrated their potential in supporting bone generation [125]. Similar results were obtained with biomineral-coated PLLA or PCL scaffolds implanted subcutaneously into mice in vivo. In direct comparison to uncoated scaffolds, there was an improved ingrowth of bone material, good bone contact with the scaffold, and a more distinct formation of bone-like tissue [126].

In contrast to the previously mentioned processes, the technique of electronic beam melting (EPB) also has to be mentioned, in which an electronic beam is directed via electromagnetic coils onto the powder layer in order to melt it down. This process enables the printing of objects with an enormously high density and corresponding mechanical strength, but is also very slow and has a lower resolution than SLM, for example. Since this method also requires a vacuum chamber to control the electronic beam without interference, it is also very expensive [127].

Basically, none of these processes are suitable for the direct printing of living cells. This is due to the actual manufacturing process. The materials used are available in powder form and sintered or melted with the addition of thermal energy and then hardened during subsequent cooling. The resulting temperatures are not suitable for processing living cells, but are instead applied for plastics, metals, or ceramics, which makes them important in dentistry, among other areas [85]. 

### 5.6. Directed Energy Deposition (DED)

**Figure 14:**
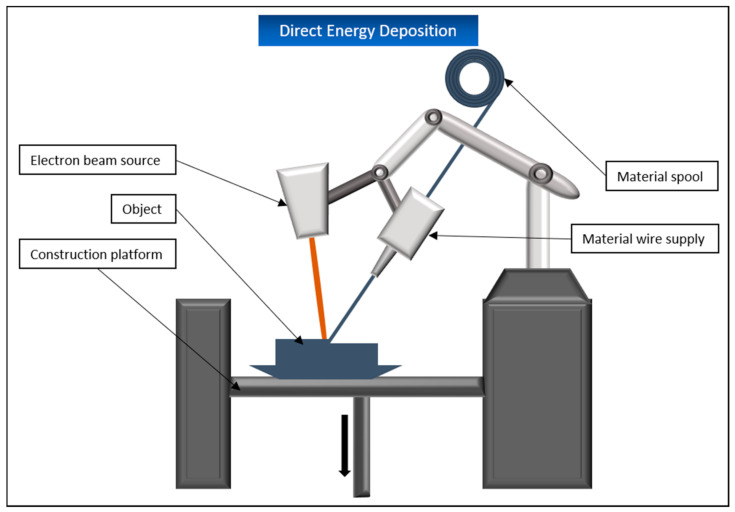
Schematic representation of the direct energy deposition. An electron beam melts the printing material, which is continuously applied layer by layer at the same time. The melted layers harden as they cool, creating the desired object.

DED, or depending on the material used or variations in the process, also known as directed light fabrication or laser engineered net shaping, represents a middle way of material extrusion and powder bed fusion. The printing material is either in the form of powder or wire and is applied layer by layer through a freely movable nozzle on the design platform. In parallel, an energy source, usually a laser or an electron beam, is used to melt the material, which is then cured by cooling (Figure 14). The materials mainly used for this process are mostly different metals, but ceramics and polymers can also be printed with this application.

DED is mainly used in aerospace, marine, or architecture. The medical sector, however, has not yet developed it to a large extent. A big advantage of the process is the possibility to repair existing components instead of printing them from scratch. It is also possible to modify the properties of individual parts by coating them. The disadvantage of this process is that the manufactured parts have a low resolution compared to other 3D printing systems and must be reworked after printing. Additionally, DED systems are still very expensive to purchase. This is partly due to the fact that laser-based variants require a chemically inert chamber to avoid unwanted reactions of the processed metal. This requires, among other things, a not inconsiderable amount of inert gas. Electron beam-based variants, in turn, require a vacuum in the design chamber, which is also a cost factor in its maintenance [128,129].

As with laser bed fusion, this process is not suitable for the printing of living cells due to its functional principle.

### 5.7. Sheet Lamination/Laminated Object Manufacturing (LOM)

Sheet lamination is used in the production of three-dimensional constructions, which are composed of sheets of the manufacturing material layered on top of each other. These materials are usually paper, plastic film, or cellulose. However, thin layers of metal can also be processed [4]. For this purpose, the materials are available as thin sheets and pre-treated on one side with an adhesive substance.

**Figure 15:**
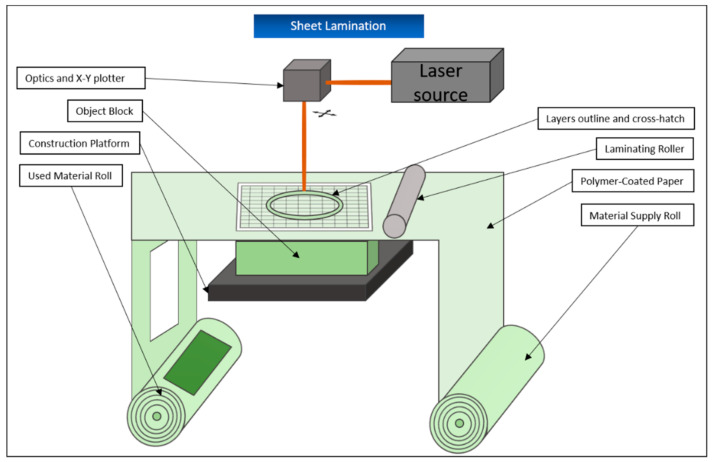
Schematic representation of the sheet lamination. The layers of the intended object are cut out of a continuously running roll of material under usage of a laser source and connected via a laminating roller.

One layer of the sheet is rolled out on the construction platform with the adhesive side down and a heated roll runs over, further attaching it to the construction platform. Then, a freely movable laser cuts the contours of the object to be created out of the layer. Afterward, the laser divides the not further required parts of the sheet around the actual object into smaller squares that are later easier to break out and serve as a supporting structure during the rest of the printing process. The construction platform is then lowered by one-layer thickness, the next layer of material is unrolled and attached to the previous one by the roller, and the process is repeated (Figure 15). At the end of the procedure, the block bonded together in this way is removed from the platform and the parts that are not needed are broken out [87,130]. The process offers the advantages of low production costs and the possibility to produce larger parts without problems. In addition, this building process creates almost no internal stresses in the later object and deformations such as shrinkage virtually do not occur. However, the manufactured objects do not have a very pronounced mechanical stability and the resolution of the process is not particularly high [4,130]. For this reason, LOM is mainly used for fast and cost-effective production of prototypes not intended for later application.

## 6. Discussion

Overall, there is neither a universally applicable 3D printing technology nor a bioink that would be suitable for every area of application. Therefore, a differentiated view of the available applications is required.

While some 3D printing processes such as SLM or binder jetting are not suitable or only suitable to a limited extent for bioprinting from the outset, other processes show very different strengths and weaknesses when used for tissue engineering. LGDW enables targeted and maximally gentle placement of individual cells. In the future, this could pave the way for new investigation approaches regarding the self-assembly of tissues or cell–cell interactions [112]. At the same time, this procedure is currently too slow to produce a high number of cells for a larger tissue in a reasonable time frame.

Methods based on the extrusion principle are faster, cheaper, and quantitatively more effective. However, this comes at the expense of tissue resolution. Additionally, the shear forces acting on the cells in the nozzle and the general viscosity of the bioink used must be considered [69,88].

In order to be able to print more complicated tissues in the future such as human skin, further improvements of both 3D printing applications and bioinks are necessary. While the first promising experiments were able to show a successful 2-layering of epidermis and dermis with and without pigmentation [6,82,107], future challenges remain, for example, in a sufficient vascularization of the tissues [2,131]. A promising approach for solving this problem in the future could be the use of so-called 4D printing, in which time is integrated as an additional factor into the printing process [132]. This is realized by the use of biomaterials, capable of changing their shape under a stimulus such as water or temperature during or after the printing process [15]. Another approach pursues the maturation of the tissue (e.g., through cellular coating or matrix deposition) after the actual printing has been completed. Both approaches seem to be promising for producing a satisfactory vascularization of artificially created tissues [133,134].

Furthermore, there is still a lack of a fast, precise and, at the same time, cell-friendly method for producing tissue with a larger area and volume. In addition, it has not yet been possible to print tissues that are completely identical to the human original. For the printing of human skin, there is still no satisfactory method for installing the tactile and vibration-sensitive units into the printed tissue.

A promising approach to solve these problems can be found in bottom-up printing. By combining different functional micro-units, consisting of different cells, to form the final macro-unit, both the printing speed and the functionality of the resulting tissue could be improved [11].

Looking at the research listed in this review, it is also noticeable that much of it deals with the general possibility of printing living cells using the individual applications and the subsequent cell viability of the printed tissues. Future research should focus more on subsequent functionalization in vitro and in vivo, as the long-term goal of bioprinting is not to print a stable tissue alone, but to make it useful, especially for individual patients.

With a view to the future of bioprinting, several interesting approaches and modifications of the previous technologies are in the focus of the efforts.

This includes, for example, the so-called “in situ-“or “in vivo-bioprinting” [135]. Instead of first producing a tissue in vitro and subsequently inserting it into the patient’s desired anatomical structure, the required tissue is printed directly into the patient’s body. While this approach avoids some of the disadvantages of conventional bioprinting such as the need for subsequent incubation of the tissue or the usually poor mechanical stability of the printed structure, which considerably complicates its inserting into the patient without damaging the construct, this technology is still in its infancy development [135]. In the past, some promising studies could be carried out with superficial or solid anatomical structures such as skin, cartilage, or bone tissue [136,137,138]. Particularly for use on deeper or softer tissues, the possibility of adequate sterilization during the printing process is required. The respective organs also have to be fixed during the printing in order to avoid errors due to the target structures slipping [135].

In the course of this review, it could be shown that the different printing technologies come with very different requirements on the biomaterials used and the result of the print depends to a large extent on different parameters, depending on the particular printing application. So far, these parameters have mainly been determined experimentally and manually calibrated by the researchers. With the help of artificial intelligence and so-called machine learning, many 3D printing processes could not only be automated in the future, but above all optimized [139,140]. Simply explained, machine learning is based on the ability of an artificial intelligence (AI) or computer system to continuously develop itself by analyzing the supplied input data, to recognize patterns and algorithms in the data, and to use this input to draw conclusions for new, unknown supplied data. The combination of machine learning with 3D printing technologies could not only improve the printing process by automatically optimizing the printing parameters, but also the pre- and post-printing processes. For example, the 3D models designed for the printer, which are generated from CT or MRT images of the patient, can be tailored much more precisely to the actually required proportions when analyzed and designed by an AI [139]. Machine learning would be able to take the greatest strength of bioprinting, the production of tissues that are optimally adapted to the individual patient, to a new level.

Considering the enormous progress of the entire 3D printing technology since its origins in the 1980s, and especially the steady progress in the field of bioprinting as well as the newly emerging technologies and solutions for the continuous improvement of the printing applications, the use of this technology as a simple and effective standard in medicine still seems a distant goal, but one that will eventually be achieved at some point.

## Figures and Tables

**Table 1:** Vat polymerization-based printing technologies in tissue engineering: Overview of recent study results. hMSC: human mesenchymal stem cell; PDLLA: poly (D, L-lactic acid); PEG: polyethylene glycol; GelMA: gelatin-methacrylamide; hADSC: human adipose-derived stem cell; MSC: mesenchymal stem cell; PEGDA: polyethylene glycol diacrylate; MSC: mesenchymal stem cells; GelMod: methacrylamide-modified gelatin; CHO: Chinese hamster ovary; SU-8: SU-8 photoresist; SLA: stereolithography; 2PP: 2-photon polymerization.

Vat Polymerization				
Printing Application	Cells	Biomaterials	Results	Reference
Stereolithography	hMSCs seeded after scaffold-printing	PDLLA-PEG-PDLLA	🡺 Creating of a biocompatible und biodegradable resin for bioprinting 🡺 Adhesion and proliferation of the seeded cells within 5 days after printing	Seck et al. [50]
	NIH 3T3 fibroblasts	PEG & GelMA	🡺 Demonstrated SLA-Printing of encapsulated cells with a cell survival of >80% after printing	Wang et al. [52]
	hADSCs	PEGDA	🡺 Demonstrated SLA-Printing of encapsulated cells with a cell survival of >90% after printing and 7 days later under cultivation	Lin et al. [53]
Two-Photon Polymerization	Porcine MSC seeded after scaffold-printing	GelMod	🡺 Demonstrated capability of 2PP to control porosity and microtopography of the printed scaffolds 🡺 Adhesion and differentiation of the seeded cells into osteogenic lineage	Ovsianikov et al. [59]
	Seeding of CHO cells, GFSHR-17 granulosa cells, GM7373 endothelial cells and SH-SY5Y neuroblastoma cells in different test setups	Ormocomp^®^ & SU8 in different test setups	🡺 Proved cell supporting properties and biocompability of Ormocomp^®^ and SU-8 as polymers for 2PP 🡺 Cells were able to grow even on vertical structures, printed with Ormocomp^®^	Ovsianikov et al. [60]

**Table 2:** Extrusion-based printing technologies in tissue engineering: Overview of recent study results. FB: fibroblast; KC: keratinocyte; PDMS: poly(dimethylsiloxane); HDF: human primary dermal fibroblasts; HEK: human primary epidermal keratinocytes; PCL: polycaprolactone; hASC: human adipose stem cells; ECM: extracellular matrix.

Extrusion-Based			
Cells	Biomaterials	Results	Reference
FBs & KCs	PDMS	🡺 Multilayered dermal/epidermal-like layers created	Lee et al. [78]
HDFs & HEKs	PCL	🡺 Created a human skin model with a functional transwell system	Kim et al. [77]
hASCs	Collagen & Alginate	🡺 Cell viability >90% of the encapsulated cells	Yeo et al. [76]
Bovine Chondrocytes	Gellan, Alginate & BioCartilage (cartilage extracellular matrix particles)	🡺 Demonstrated the capability of reconstituting ECM particles into de novo bioprinted structures	Kesti et al. [79]
HUVECs & HEK293	Gelatin & PEO (polyethylene oxide)	🡺 Created a bioink applicable for entrapping and printing cells in freeform fabrication	Irvine et al. [80]
hASCs	Collagen/ECM & alginate	🡺 Sufficient viability and differentiation of cells with this novel bioprinting approach	Lee et al. [81]
Primary human fibroblasts & keratinocytes from human skin biopsies	Human Plasma	🡺 Printed tissue very similar to human skin and indistinguishable from manually produced two-layer dermo-epidermal models	Cubo et al. [82]

**Table 3:** Material jetting—based printing technologies in tissue engineering: Overview of recent study results. AFS: amniotic fluid-derived stem cells; CHO: Chinese hamster ovary; HMVEC: human microvascular endothelial cell; PEG: polyethylene glycol; BM-MSC: bone marrow-derived mesenchymal stem cells; hESC: human embryonic stem cell; hiPSC: human induced pluripotent stem cell; NHDF: neonatal human dermal fibroblast; NHEK: neonatal human epidermal keratinocytes; FB: fibroblast; KT: keratinocyte; hMSC: human mesenchymal stem cell; PCL: polycaprolactone; LIFT: laser-induced forward transfer; LAP: laser-assisted printing; LGDW: laser-guidance direct writing.

Material Jetting				
Printing Application	Cells	Biomaterials	Results	Reference
Inkjet-based				
Thermal inkjetting	AFS	Collagen & Alginate	🡺 Successful construction and in vivo implantation with differentiation into a bone-like tissue	De Coppi et al. [96]
	CHOs and embryonic rat motoneurons	“biopaper”, consisting of soy agar and collagen hydrogels	🡺 Demonstration of thermal inkjetting ability to print with cell loss of less than 10%.	Xu et al. [97]
	HMVECs	Fibrin	🡺 Microvascular structures with moderate integrity printed	Cui et al. [113]
Piezoelectric	Fibroblasts	Collagen & Alginate	🡺 Successful printing of vascular structures with horizontal and vertical bifurcation	Christensen et al. [114]
	3T3 mouse fibroblasts	Sodium Alginate	🡺 study of the effect of cell concentration in the bioink on the droplet formation process	Xu et al. [98]
Acoustic wave jetting	Mouse embryonic stem cells, fibroblasts, AML-12 hepatocytes, human Raji cells & HL-1 cardiomyocytes	Various biofluids, including agarose hydrogels	🡺 creation of a new single cell acoustic picolitre droplet ejector & printing of various encapsulated cells in picolitre droplets with viabilities above 89,8%	Demirci et al. [99]
Microvalve-based	Fibroblasts & Keratinocytes	Collagen hydrogel	🡺 Construction of a soft tissue comparable in cell density, morphology and thickness to in vivo human skin	Lee et al. [6]
	Fibroblasts, Keratinocytes were subsequently seeded onto the printed construct	PEG	🡺 Production of skin-like soft tissue models	Rimann et al. [115]
	AFS, BM-MSCs	Fibrin-collagen gel	🡺 AFS- and MSC-cell-treated wounds showed increased wound closure and re-epithelialization compared to “cell-free” fibrin-collagen-gel treated wounds	Skardal et al. [116]
	hESCs & hiPSCs	Alginate hydrogel	🡺 Demonstration of printing hiPSCs and hESCs without affecting their viability and pluripotency 🡺 directing differentiation into specific lineages was also shown	Faulkner-Jones et al. [117]
Unassignable	HMVEC & NHDF, NHEKs were added subsequently after printing	Comparison of Apligraf^®^ and printed skin graft	🡺 Accelerated wound healing and formation of new microvessels within the bioprinted graft, compared to the other grafts	Yanez et al. [118]
Laser-assisted				
LIFT	NIH 3T3 fibroblasts	Sodium alginate & calcium chloride	🡺 Less gelation time of printed cells increases post-transfer viability 🡺 Cell viability after 24 h incubation decreases as laser fluence or alginate concentration increases	Gudapati et al. [119]
	NIH3T3 FBs, HaCat KTs & hMSCs	Collagen	🡺 Printed multicellular constructs with micrometer accuracy 🡺 LIFT does not change phenotype of transferred hMSC & maintains their ability to differentiate	Koch et al. [109]
	MG 63 cells	Alginate, PCL electrospun scaffold	🡺 Demonstrated superiority of layer by layer bioprinted tissues in maintaining cell viability in vitro and enhancing cell proliferation in vivo compared to “conventional scaffold seeding”	Catros et al. [120]
	Fibroblasts & Keratinocytes	Matriderm H (bovine collagen/elastin contents)	🡺 Successful 3D printing of cell construct via LAP and subsequent tissue formation in vivo	Michael et al. [108]
LGDW	Embryonic chicken spinal cord cells	Deposition on a glass target surface	🡺 demonstration of precise cell placement via LGDW 🡺 placed cells showed viable after influence of laser light	Odde et al. [111]
	HUVECs	Collagen gel or Matrigel	🡺 demonstrated ability of patterning cells with micrometer precision 🡺 direct formation of vascular structures in vitro and an aggregate with liver sinusoid-like organization	Nahmias et al. [112]
